# Effect of intravitreal or sub-tenon triamcinolone acetonide injection at completion of vitrectomy on peripheral retinochoroidal thickness in eyes with proliferative diabetic retinopathy

**DOI:** 10.1038/s41598-018-37220-3

**Published:** 2019-01-17

**Authors:** Yoshito Fujiwara, Takeshi Iwase, Kentaro Yamamoto, Yoshitaka Ueno, Eimei Ra, Hiroko Terasaki

**Affiliations:** Department of Ophthalmology, Nagoya University Hospital, Nagoya, Japan

## Abstract

The effect of triamcinolone acetonide (TA) on the peripheral retinochoroidal thickness was determined after pars plana vitrectomy (PPV) with scatter photocoagulation in eyes with proliferative diabetic retinopathy. The peripheral retinochoroidal thickness was measured at 5 mm from the limbus in the four quadrants using anterior segment optical coherence tomography before, and 3 days, and 1 and 2 weeks after the surgery. The total peripheral thickness was significantly thicker than the baseline thickness after PPV alone (*P* < 0.001; 18 eyes), PPV combined with intravitreal TA injection (IVTA; *P* = 0.011; 19 eyes), and PPV combined with sub-tenon TA injection (STTA; *P* = 0001; 23 eyes). The total peripheral thickness in the PPV group at 3 days after surgery was significantly thicker than that of the PPV + IVTA (*P* = 0.015) and of the PPV + STTA groups (*P* = 0.016). Multiple linear regression analyses showed that the injection of TA by the two routes and the number of photocoagulation burns were significantly correlated with the total peripheral thicknesses at 3 days after the surgery. The results indicate that the PPV with large number of intraoperative scatter photocoagulation burns caused an increase in the total peripheral thickness and an administration of either IVTA and STTA can reduced the degree of thickening.

## Introduction

Proliferative diabetic retinopathy (PDR) is one of the leading causes of blindness in the working population^1^. Panretinal laser photocoagulation (PRP) is an effective treatment for PDR, and its application has been shown to reduce the risk of a severe reduction of vision^2–4^. Pars plana vitrectomy (PPV) is also used to treat PDR, and its effectiveness is due to its removal of vitreous opacities and reduction of retinal traction. Therefore, it has been recommended that PRP should be performed during the vitrectomy if it had not been performed before the PPV. This treatment protocol will reduce the risk of the onset and progression of neovascularization and rubeotic glaucoma. However, PRP has complications, e.g. intraocular pressure (IOP) elevation, massive choroidal detachment, and angle closure glaucoma^5^. In addition, peripheral choroidal detachments can frequently occur after PPV combined with PRP^6^ and also after PRP alone^7^.

Silicone oil and gas tamponades have been shown to be helpful in treating eyes with severe PDR^8^. However, it has been reported that PRP combined with such tamponade on eyes with PDR has a high risk of elevating the IOP^9^, and Yamamoto *et al*. have reported that a large number of intraoperative scatter photocoagulations can cause a thickening of the retina and choroid which then leads to a reduction in the volume of the vitreous cavity^6^. These changes can then cause an elevation of the IOP in the early postoperative period in eyes with PDR^6^. Thus, suppression of the thickening and the postoperative IOP elevation is critical for patient care in eyes with PDR undergoing PPV.

Corticosteroids have been used to treat inflammations due to their ability to suppress leucocyte migration, reduce cytokine production, and antagonize the action of vascular endothelial growth factor (VEGF)^10,11^. Three potent synthetic corticosteroids, triamcinolone acetonide (TA)^12–15^, dexamethasone^16^, and fluocinolone acetonide^17^, have been used to treat diabetic macular edema (DME). There are two routes for the administration of TA, viz., an intravitreal and a sub-tenon route, and the effectiveness of intravitreal TA (IVTA)^12,13^ and sub-tenon TA (STTA)^14,15^ injections on the DME have been reported. The question then arises on whether it will be possible to suppress the thickening of the peripheral retinochoroidal tissue in eyes that have undergone PRP during PPV.

Thus, the purpose of this study was to evaluate the effect of an IVTA or STTA injections on the peripheral retinochoroid thickness after PPV with intraoperative complete scatter photocoagulation.

## Results

### Demographics and surgical characteristics of patients

The demographics and surgical characteristics of the patients are shown in Table 1. Seventy-six eyes of 76 patients underwent 23- or 25-gauge PPV combined with scatter photocoagulation with or without an IVTA or STTA injection for PDR and were examined. Of these, 16 eyes were excluded: 4 eyes for fewer than 500 burns, 1 eye for burns limited to one quadrant, 2 eyes for silicon oil tamponade, and 9 eyes that did not have all of the examinations. In the end, there were 18 eyes of 18 PDR patients that underwent PPV without TA (PPV group), 19 eyes of 19 PDR patients that underwent combined PPV with IVTA (PPV + IVTA group), and 23 eyes of 23 PDR patients that underwent PPV with STTA (PPV + STTA group). The differences in the patient demographics and the surgical characteristics were not significant among the three groups.

**Table 1:** Patient demographics and surgical characteristics.

Characteristic	PPV	PPV + IVTA	PPV + STTA	*P* value
n (eyes)	18	19	23	—
Age (years)	61.8 ± 10.9	59.8 ± 11.3	57.0 ± 14.1	0.426
Men/Women	11/7	13/6	15/8	0.900
Axial length (mm)	24.03 ± 1.16	23.86 ± 1.01	23.85 ± 0.94	0.872
Mean periperal RCT before surgery (µm)	201.9 ± 45.2	202.8 ± 11.9	213.4 ± 25.8	0.395
PPV/PPV + IOL	10/8	8/11	10/13	0.781
23/25 gauge	4/14	1/18	1/22	0.175
Photocoagulation burns	1645 ± 818	1164 ± 573	1699 ± 712	0.068
Operation time (min)	87.3 ± 30.0	64.4 ± 46.2	62.83 ± 31.1	0.064
None/Air or SF6	6/12	5/14	10/13	0.513

PPV: pars plana vitrectomy, PEA: phacoemulsification and aspiration, IOL: intra-ocular lens, SF6: sulfur hexafluoride.

### Changes in total peripheral and retinochoroidal thicknesses

**Figure 1:**
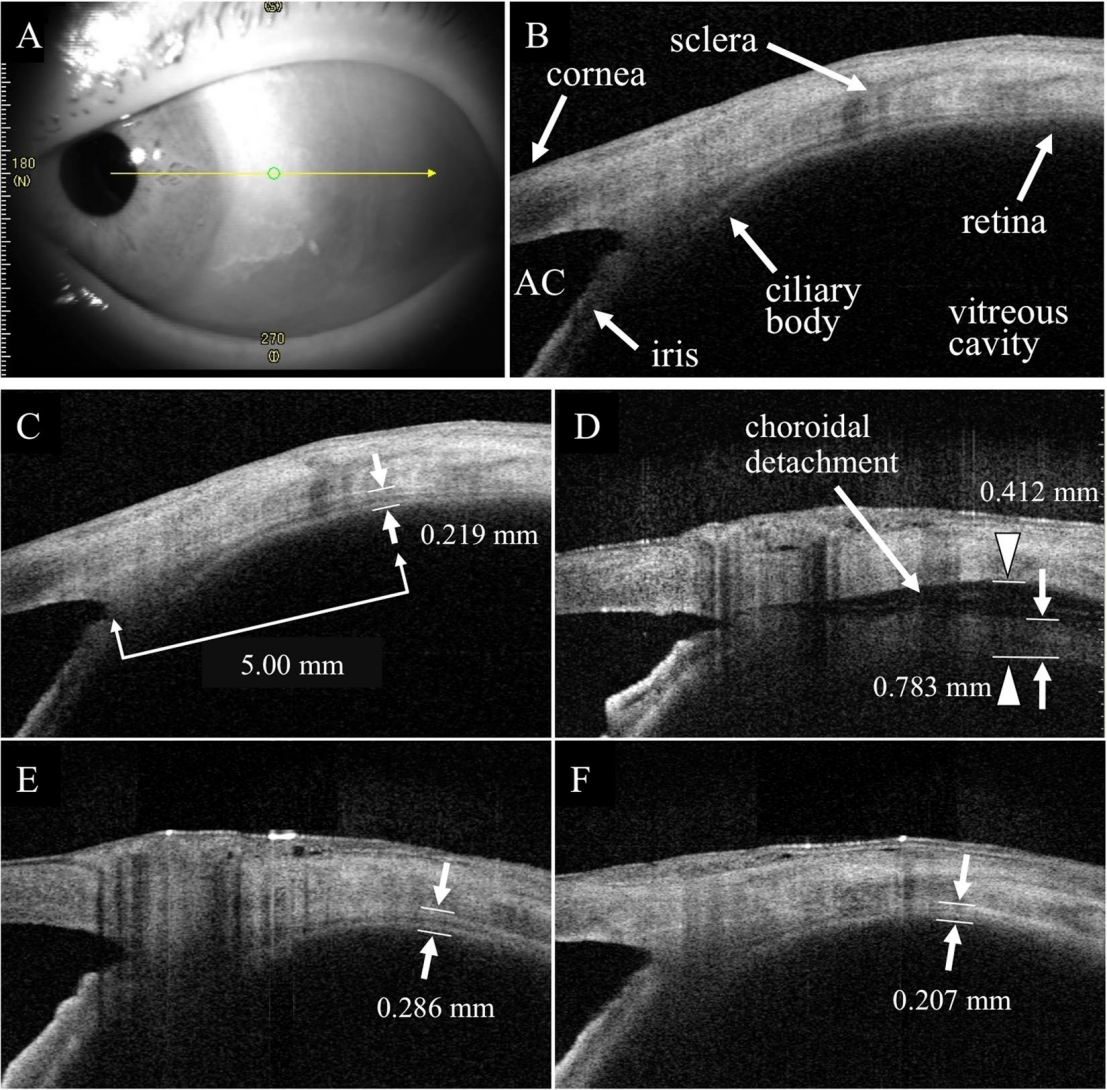
Representative anterior segment optical coherence tomographic (OCT) images of eyes with proliferative diabetic retinopathy (PDR) that underwent pars plana vitrectomy (PPV) without an administration of triamcinolone acetonide (TA). An anterior segment optical coherence tomographic (OCT) image of the temporal area of the retina. (**A**) The structure was determined in an image taken by anterior segment-OCT. (**B**) The vertical retinochoroidal thickness was manually measured from the vitreoretinal interface to the outer surface of the choroid at 5000 µm from the limbus in the four quadrants in the anterior segment-OCT images. (**C**–**F**) Both the retinochoroid thickness and the distance from the outer surface of the choroid to the inner surface of the sclera as the choroidal detachment thickness were measured separately in eyes with a retinochoroidal detachment. The white arrowheads indicate the total peripheral thickness consisting of the retinochoroid thickness (white arrow) plus the height of the choroidal detachment 3 days after surgery. (**D**) The retinochoroid thickness is decreased and the choroidal detachment is not present at 1 week (**E**) and 2 weeks (**F**) after the surgery. AC, anterior chamber.

The mean total peripheral thickness, the mean retinochoroidal thickness, and the mean thickness of the choroidal detachment before and after surgery are shown in Table 2. In the PPV group, the mean total peripheral thickness, the mean retinochoroidal thickness, and the mean thickness of the choroidal detachment were significantly thicker than that before surgery (*P* < 0.001, *P* < 0.001, *P* < 0.011, respectively; Fig. 1).

**Table 2:** Changes in the total peripheral thickness, the retinochoroidal thickness, thickness of choroidal detachment, and intraocular pressure.

Group	Thickness	Before surgery	Day 3	Week 1	Week 2	P-value
PPV	Total peripheral thickness (µm)	202 ± 45	5434 ± 338	269 ± 163	223 ± 68	<0.001
	Retinochoroidal thickness (µm)	202 ± 435	392 ± 179	234 ± 67	210 ± 45	<0.001
	Height of choroidal detachment (µm)	0	152 ± 188	35 ± 107	12 ± 35	<0.001
	IOP	14.52 ± 3.2	13.3 ± 2.4	12.8 ± 3.1	13.2 ± 3.3	0.492
PPV + IVTA	Total peripheral thickness (µm)	202 ± 11	343 ± 161	203 ± 14	181 ± 9	0.011
	Retinochoroidal thickness (µm)	202 ± 11	267 ± 62	201 ± 10	181 ± 9	0.005
	Height of choroidal detachment (µm)	0	76 ± 102	2 ± 6	0	0.022
	IOP	14.4 ± 3.6	13.6 ± 3.4	15.01 ± 3.3	15.12 ± 3.0	0.909
PPV + STTA	Total peripheral thickness (µm)	213 ± 26	372 ± 180	240 ± 33	235 ± 68	<0.001
	Retinochoroidal thickness (µm)	213 ± 26	289 ± 83	233 ± 34	223 ± 33	<0.001
	Height of choroidal detachment (µm)	0	82 ± 125	1 ± 5	0	0.003
	IOP	13.57 ± 2.2	13.07 ± 3.8	13.9 ± 3.2	15.0 ± 3.2	0.189

PPV: pars plana vitrectomy, IVTA: intravitreal triamcinolone acetonide, STTA: sub-tenon triamcinolone acetonide

**Figure 2:**
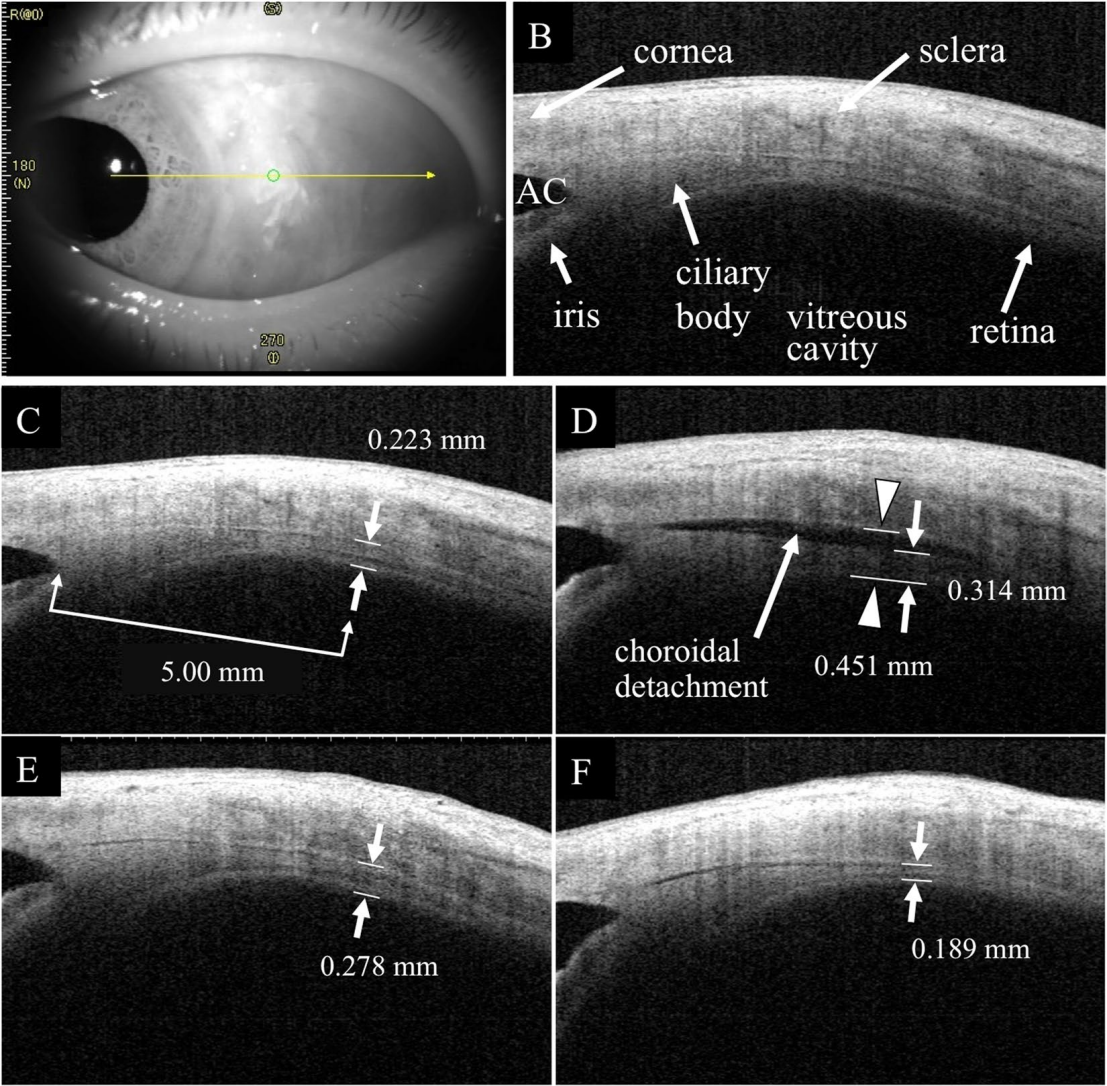
Representative anterior segment-OCT images of eyes with PDR that underwent PPV with an intravitreal triaminocolone acetomide (IVTA) injection. An anterior segment-OCT image was taken of the temporal area. (**A**) The retinochoroidal thickness was measured in eyes with PPV + IVTA. (**B**–**E**) The total peripheral thickness was thinner in the PPV + IVTA group than that in the PPV group. AC, anterior chamber.

**Figure 3:**
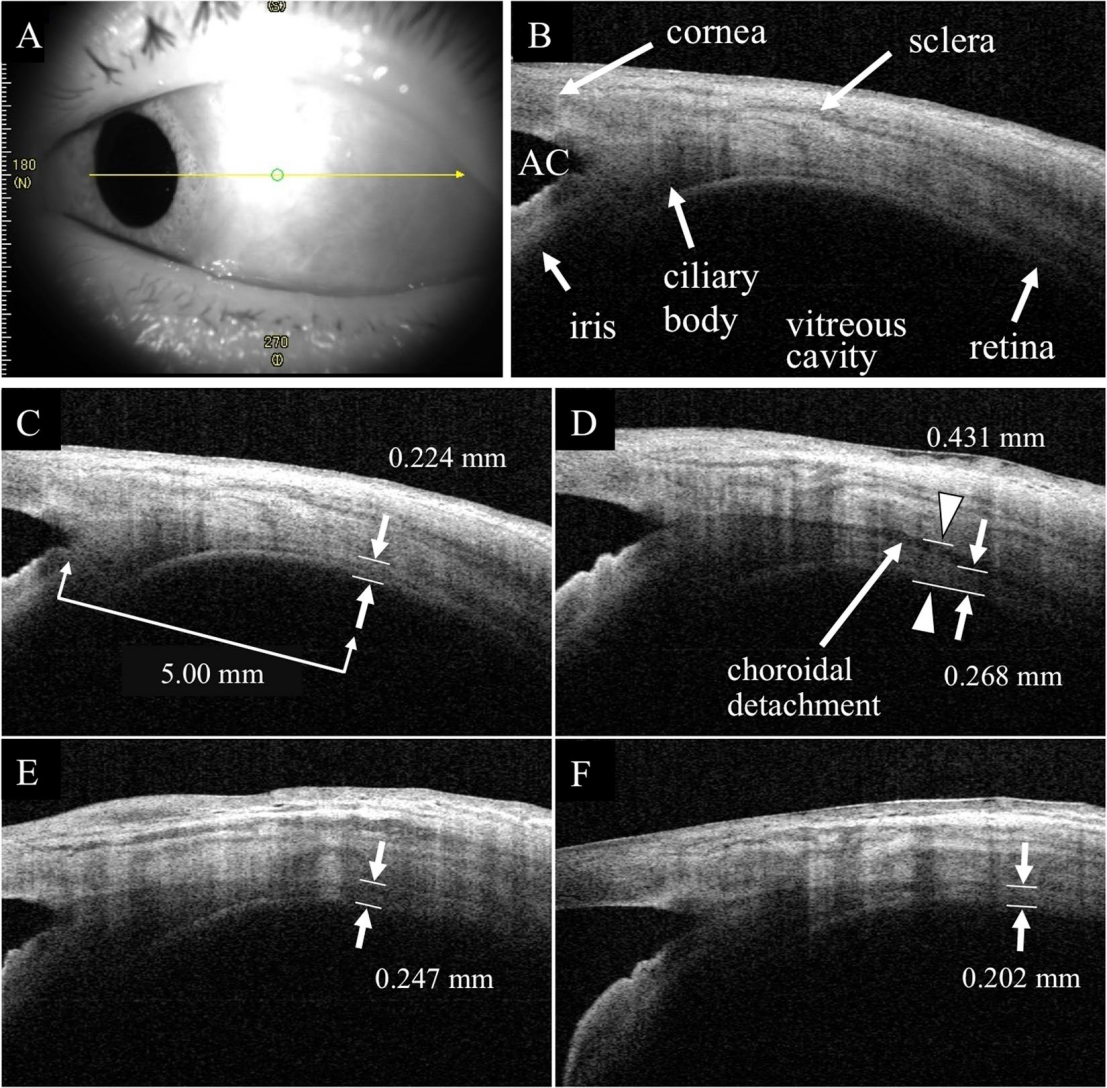
Representative anterior segment-OCT images of eyes with PDR that underwent PPV with sub-tenon TA (STTA) injection. An anterior segment-OCT image was taken at the temporal area. (**A**) The retinochoroidal thickness was measured in eyes with PPV + STTA. (**B**–**E**) The total peripheral thickness was thinner in the PPV + STTA group than that in PPV group. AC: anterior chamber.

In the PPV + IVTA group, the mean total peripheral thickness, the mean retinochoroidal thickness, and the mean thickness of the choroidal detachment were significantly thicker than that before the surgery (*P* = 0.011, *P* = 0.005, *P* = 0.022, respectively; Fig. 2). In the PPV + STTA group, the mean total peripheral thickness, the mean retinochoroidal thickness, and the mean thickness of the choroidal detachment were significantly thicker than that before surgery (*P* < 0.001, *P* < 0.001, *P* = 0.003, respectively; Fig. 3).

**Figure 4:**
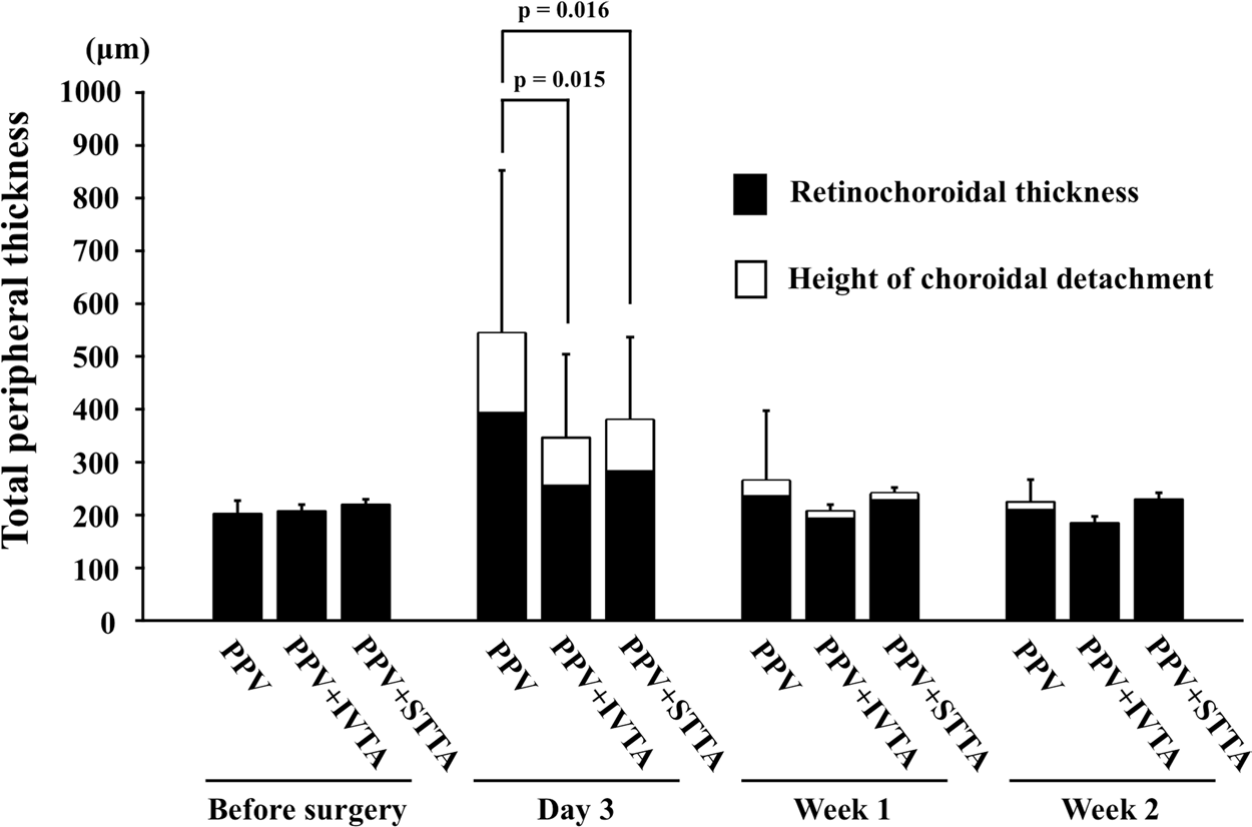
Changes in the mean total peripheral thickness. There was a significant difference in the total peripheral thickness between the PPV and the PPV + IVTA groups (*P* = 0.015) or the PPV and the PPV + STTA groups (*P* = 0.016) only at 3 days after surgery.

The mean increase of the total peripheral thickness 3 days after surgery was 341 ± 317 µm in the PPV group, 141 ± 166 µm in the PPV + IVTA group, and 158 ± 182 µm in the PPV + STTA group. The increase in the thickness in the PPV group was significantly greater than that in the PPV + IVTA group (*P* = 0.015) and in the PPV + STTA group (*P* = 0.016) at only 3 days after the surgery (Fig. 4).

### Choroidal detachments after surgery

Choroidal detachments were present in 14 of 22 eyes (77.8%) in the PPV group, in 9 of 19 eyes (47.4%) in the PPV + IVTA group, and in 13 of 24 eyes (56.5%) in the PPV + STTA group at 3 days after the surgery (Table 3). The choroidal detachment disappeared with time in all of the three groups but persisted in 4 eyes in the PPV group at 2 weeks after surgery. None of the eyes had a choroidal detachment at 2 weeks after the surgery in the other two groups. The proportion of eyes with a postoperative choroidal detachment in the PPV group was significantly higher than that in the PPV + IVTA and PPV + STTA groups at 2 weeks after the surgery (*P* = 0.012).

**Table 3:** Proportion of postoperative choroidal detachment.

Time	Postoperative choroidal detachment			
	PPV (n = 18)	PPV + IVTA (n = 19)	PPV + STTA (n = 23)	*p*-value
Day 3	14 (77.8%)	9 (47.4%)	13 (56.5%)	0.151
Week 1	3 (16.7%)	1 (5.3%)	2 (8.7%)	0.542
Week 2	4 (22.2%)	0 (0%)	0 (0%)	0.012

PPV: pars plana vitrectomy, PEA: phacoemulsification and aspiration, IOL: intra-ocular lens.

### Differences in changes of total peripheral thickness in four quadrants

**Figure 5:**
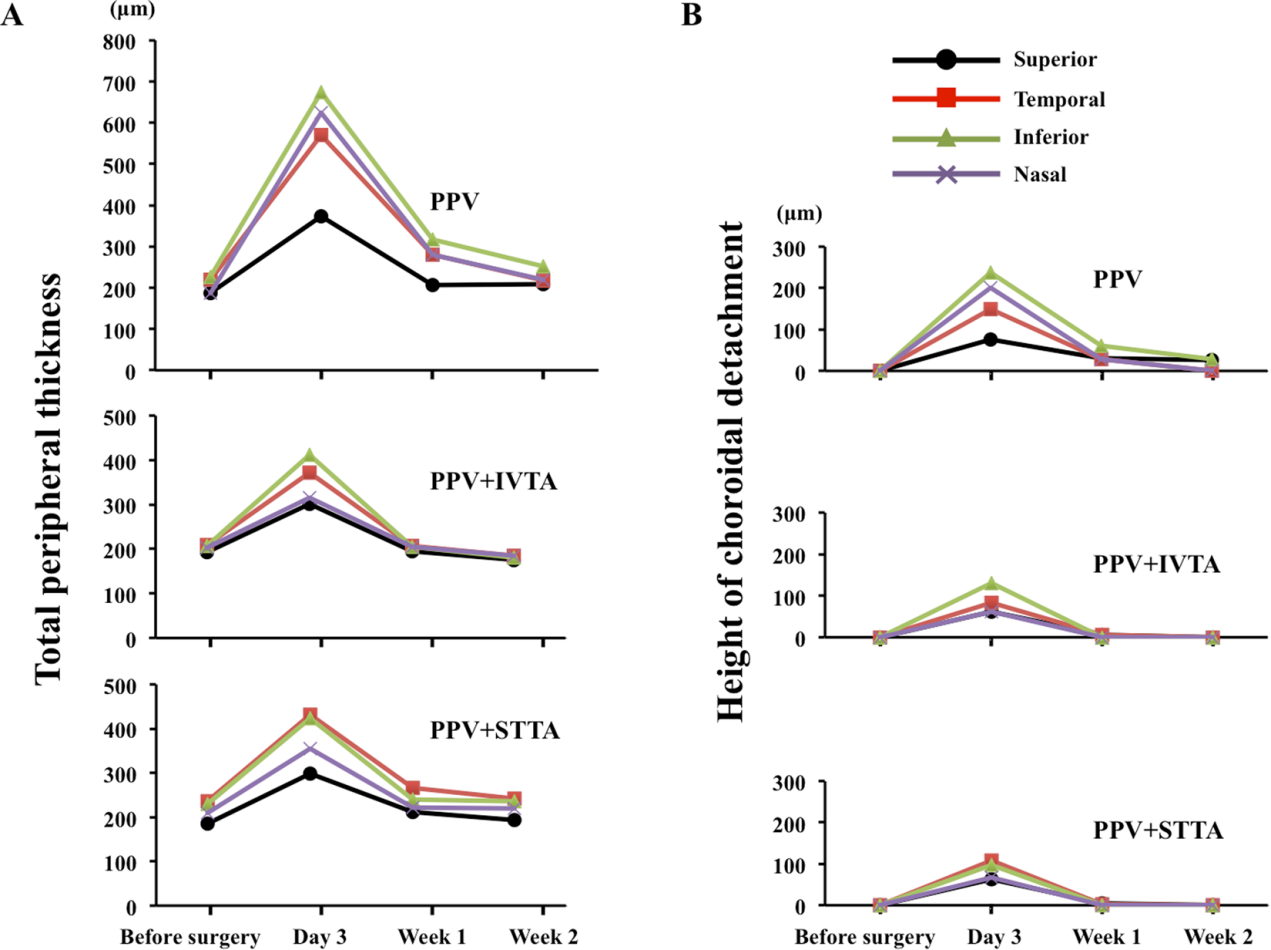
Changes in the mean total peripheral thickness in each quadrant. The total peripheral thickness of the inferior peripheral quadrant was significantly thicker than the other quadrants at 3 days (*P* < 0.001) and at 1 week (*P* = 0.016) after surgery in the PPV group. (**A**) The thickness of the choroidal detachment in the superior peripheral quadrant was significantly less than that in the other quadrants only at 3 days (*P* = 0.046) after the surgery in the PPV group (**B**).

The total peripheral thickness of the peripheral inferior quadrant was significantly thicker than the other three quadrants at 3 days (*P* < 0.001) and at 1 week (*P* = 0.016) after surgery in the PPV group. However, the total peripheral thickness was not significantly different among the quadrants in the PPV + IVTA and the PPV + STTA groups (Fig. 5A). The thickness of the choroidal detachment in the superior peripheral quadrant was significant thinner than that in the other quadrants only at 3 days (*P* = 0.046) after the surgery in the PPV group, but there were no significant differences in the thickness among the quadrants in the IVTA and STTA groups at any postoperative period (Fig. 5B).

### Correlations between changes of total peripheral thickness and number of photocoagulation burns

**Figure 6:**
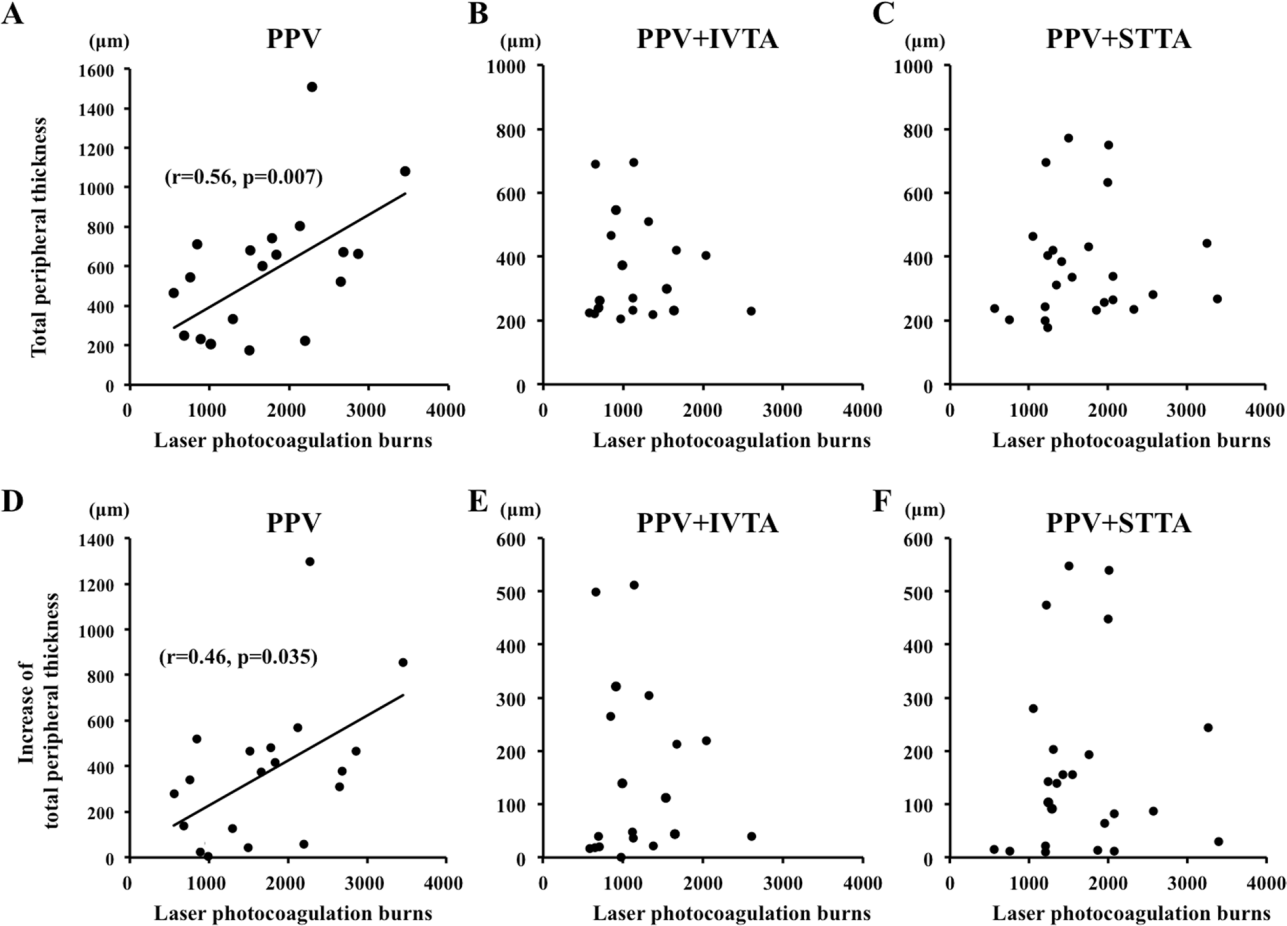
Scatterplot of the total peripheral thickness versus the number of laser photocoagulation burns. The total peripheral thickness was significantly and positively correlated with the number of laser photocoagulation burns (r = 0.56, *P* = 0.007) in the PPV group (**A**) but was not significantly correlated in the PPV + IVTA (**B**) and PPV + STTA groups. (**C**) The increase of the total peripheral thickness was significantly and positively correlated with the number of laser photocoagulation burns (**D**) (r = 0.46, *P* = 0.035), but was not significantly correlated with the PPV + IVTA (**E**) and PPV + STTA groups (**F**).

The results of the multiple linear regression analysis for the total peripheral thickness at 3 days after surgery are shown for all of the subjects in Table 4. The injections of TA by both routes and the number of laser photocoagulation burns were significantly correlated with the total peripheral thickness at 3 days after the surgery (*P* = 0.014, *P* = 0.017, respectively). However, the age, the operation time, and the IOP were not significantly different. The number of photocoagulation burns was significantly and positively correlated with the total peripheral thickness (r = 0.57, *P* = 0.007; Fig. 6A) and also with the increase of the total peripheral thickness at 3 days after the surgery (r = 0.54, *P* = 0.011; Fig. 6B). However, there was no significant correlation between the total peripheral thickness at 3 days after surgery and other variables including the number of laser photocoagulation burns in the PPV + IVTA and PPV + STTA groups.

**Table 4:** Results of muliple regression analysis of factors independently contributing to the total peripheral thickness.

Explanatiry variables	Coefficients	*t* Value	*P* Value
Administration of TA	−0.311	−2.529	0.014
Laser photocoagulation burns	0.303	2.466	0.017
Age (year)	−0.240	−1.949	0.057
Preoperative retinochoroidal thickness (µm)	0.225	1.805	0.077
Operation time (min)	0.114	0.800	0.427
IOP (Day 3) (mmHg)	−0.071	−0.574	0.568
Axial length (mm)	−0.038	−0.301	0.765

### Changes of IOP

There were no significant changes in the IOPs before and at any time after surgery in the three groups. In addition, there were no significant differences in the IOP in any time among the three groups.

## Discussion

Our results showed that the total peripheral thickness was significantly increased over the pre-treatment values in the PPV and PPV + STTA groups at 3 days and 1 week after the surgery. The increase of the total peripheral thickness was significantly less in the PPV + IVTA and PPV + STTA groups than in the PPV group only at 3 days after surgery. The proportion of eyes with a choroidal detachment in the PPV group at 2 weeks after surgery was significantly higher than in the PPV + IVTA and PPV + STTA groups. Multiple linear regression analyses showed that the administration of TA and the number of laser photocoagulation burns were significantly and positively correlated with the total peripheral thickness at 3 days after the surgery.

A significant thickening of the total peripheral thickness was observed after the vitrectomy with laser photocoagulation in the PPV group. In addition, the number of laser photocoagulation burns was significantly correlated with the total peripheral thickness at 3 days after surgery in the PPV group. These findings indicate that the laser photocoagulation caused the thickening of the total peripheral thickness. Gentile *et al*. examined eyes by ultrasound biomicroscopy and found that a larger number of laser burns was associated with an increase in the likelihood of choroidal effusion^18^.

Performing photocoagulation to the ischemic areas of the peripheral retina during vitrectomy is necessary to reduce the risks of postoperative complications. However, laser photocoagulation may be a significant factor in inducing postoperative inflammation^19,20^. Inflammation is a non-specific response to surgical invasion and laser photocoagulation. It has been reported that photocoagulation can cause massive choroidal detachment^5^ and transient retinal edema at the site of the photocoagulation^21^. In addition, thermal burns induce transient elevations of inflammatory cytokines in the vitreous and aqueous humor, and these cytokines can enhance the development of inflammation^22^. These results suggest that laser photocoagulation can cause not only inflammation of the retina and the choroidal capillary network^19,20,23^, but can also cause retinochoroidal detachments^6^.

It has been reported that IVTA administration combined with PRP in rabbits reduced the expressions of VEGF and proinflammatory cytokines, whereas bevacizumab prevented VEGF only^24^. These findings suggested that TA can affect different pathways with inhibitory action against anti-inflammatory and VEGF expression. In addition, steroid administration has the potential of protecting the disruption of the blood:ocular barrier in patients with PDR^24^.

The multiple linear regression analyses showed that the administration of TA by both routes was significantly associated with a decrease in the total peripheral thickness at 3 days after the surgery. The increase of total peripheral thickness in the PPV + IVTA and PPV + STTA groups at 3 days after surgery was less than that in the PPV group. The incidence of choroidal detachment in the PPV + IVTA and PPV + STTA group at 2 weeks after surgery was less than that in the PPV group. In addition, there was no significant correlation between the number of laser burns and total peripheral thickness in the PPV + IVTA and PPV + STTA groups. These results indicate that the administration of TA can inhibit the thickening of the total peripheral thickness after surgery even if the number of laser spots was relatively large. Alibet *et al*. reported that the preoperative anti-inflammatory treatment by TA administration in rhegmatogenous retinal detachment complicated by choroidal detachment resulted in a restoration of the anatomical position of the ciliary body and a significant reduction in ciliary body edema^25^. In addition, the preoperative treatment of such eyes with IVTA combined with an expansive gas tamponade especially in severe cases or alone has a number of advantages^26^. These results suggest that an administration of TA suppresses the thickening of the total peripheral thickness.

Yamamoto *et al*. compared the changes in the total peripheral thickness between PDR and ERM after vitrectomy and reported less thickening of the peripheral choroidal thickness in eyes with ERM as the mean increase of the total peripheral thickness 3 days after surgery was 132 µm in the ERM group^6^. In our study, the mean increase of the total peripheral thickness 3 days after surgery was, 141 µm in the PPV + IVTA group and 158 µm in the PPV + STTA group. We cannot compare those results directly, but they indicate that the administration of TA by both routes can suppress the thickening close to that in eyes with ERM after vitrectomy.

There was no significant difference in the increase of the total peripheral thickness between the PPV + IVTA and the PPV + STTA groups. Bonini-Filho *et al*. reported that the postoperative changes in the central macular thickness and visual acuity observed suggested that IVTA may be more effective than STTA for the management of refractory diffuse DME^27^. It appears that 4 mg of IVTA administration and 40 mg STTA administration have similar effect in terms of suppressing the thickening of the total peripheral thickness.

There have been reports that an early postoperative rise in the IOP was due to several factors including pupillary block, inflammation, migration of silicone oil into the anterior chamber, and others^28^. Laser photocoagulation can lead to a reduction in the volume of the vitreous cavity which would then cause an IOP elevation which would be an early postoperative complication in eyes with PDR, especially in eyes with a full silicone oil tamponade. The reduced compressibility of silicone oil tamponade would be able to cause an IOP elevation as an early postoperative complication. Therefore, it is important to avoid this complication not only by injecting a lesser amount of SO^6^, but also to suppress a thickening of the peripheral choroidal thickness by other methods.

An administration of either of IVTA and STTA suppressed the thickening of the total peripheral thickness. In fact, our results showed that the IOP did not increase after the administration of IVTA and STTA, and none of the eyes had an uncontrollable IOP elevation at 2 weeks after surgery. However, an administration of corticosteroids has a high risk of IOP elevation. It has been reported that the IOP was increased at 1 month postoperative period^29^, and the half-time of TA was 18.6 days in the non-vitrectomized eyes and 3.2 days in the vitrectomized eyes^30^. Because we measured the IOP at 2 weeks after the administration, and we do not know the value of the IOP after 2 weeks of the administration. Thus, we need to pay attention for the IOP in the later postoperative stage.

Our findings showed that the total peripheral thickness in the inferior quadrant was thicker than the other quadrants in the PPV group at 3 days after surgery. This indicated that the retinochoroidal detachment after surgery was not uniform in all quadrants in the PPV group and was more extensive inferiorly probably due to gravity and pooling in the inferior quadrant. However, there were no significant differences in the total peripheral thickness among the four quadrants in the PPV + IVTA and PPV + STTA groups. An administration of TA suppressed the thickening and there were no significant differences in the thickness among the quadrants.

Our study has several limitations. First, our study was a retrospective study with a small sample size. Second, the surgical procedures, e.g. operation time and types of surgery performed, and the age were different among the groups, although there were no significant differences. However, the possibility cannot be eliminated that the differences in the surgical procedures and the age may have affected the results. Third, we enrolled some PDR patients with vitreous hemorrhage, and thus the macula choroidal thickness could not be determined before the surgery and how the thickness changed after the surgery. Fourth, we measured the retinochoroidal thickness at 5 mm posterior from the limbus where we could identify the peripheral retinochoroidal thickness and the height of choroidal detachment separately. The detachment thickness might be thicker at more posterior sites but it was difficult to determine the thickness there because the eyelids prevented the measurements by anterior segment OCT. Fifth, laser photocoagulation was not performed uniformly in all of 4 quadrants. However, eyes that had burns limited to one quadrant were excluded. Accordingly, those influences should be small among the 4 quadrants. Further prospective studies on a larger number of cases with similar operation times and surgical procedures will be necessary to determine the cause of the increased retinochoroidal thickness and IOP elevation after surgery.

We conclude that a large number of intraoperative scatter photocoagulation caused the thickening of the total peripheral thickness and the reduction of volume of the vitreous cavity. An administration of either of IVTA and STTA can suppress the thickening and has a possibility to avoid an IOP elevation caused by the reduction of volume of the vitreous cavity in the early postoperative stage in PDR cases.

## Patients and Methods

### Ethics statement

This was a retrospective, observational comparative, single-center study, and the procedures were approved by the Institutional Review Board and the Ethics Committee of the Nagoya University Graduate School of Medicine. The procedures used conformed to the tenets of the Declaration of Helsinki.

#### Subjects

We reviewed the medical records of all patients who had undergone 23- or 25-gauge PPV combined with scatter photocoagulation with or without an IVTA or STTA injection for PDR at the Nagoya University Hospital from November 2013 to December 2016. All patients signed an informed consent form before surgery. The exclusion criteria included eyes that had fewer than 500 burns or burns limited to one quadrant, silicon oil tamponade, and axial length (AL) < 22 mm or > 26.5 mm. All patients underwent a comprehensive ophthalmic examination including the measurements of the IOP and axial length, slit-lamp examination, fundus examination, and optical coherence tomography (OCT) before and 3 days, and 1 and 2 weeks after the surgery.

#### Peripheral retinochoroidal thickness measurements using swept source anterior segment optical coherence tomography (AS-OCT)

We measured the peripheral retinochoroidal thickness in the images obtained by an anterior segment swept-source OCT device (CASIA SS-1000^®^, Tomey Corporation, Nagoya, Japan; Fig. 1). We instructed the patients to turn their eye to 4 directions to take the AS-OCT images to be vertical to the vitreoretinal surface at 5000 µm from the limbus without mydriasis. The vertical retinochoroidal thickness was manually measured as the distance from the vitreoretinal surface to the outer surface of the choroid at a point 5000 µm from the limbus in the four quadrants. This was done to avoid measuring different tissues in the different quadrants because the ciliary body is positioned slightly eccentrically (Fig. 1). In eyes with a choroidal detachment at the measurement region, the thickness of the choroidal detachment was measured as the distance from the outer surface of choroid to the inner surface of the sclera. The total peripheral thickness was then calculated as shown below (Fig. 1).

Total peripheral thickness = retinochoroidal thickness + choroidal detachment thickness.

### Surgical technique

After retrobulbar anesthesia with 2.5 ml of 2% lidocaine and 2.5 ml of 0.5% bupivacaine, standard 3-port PPV was performed with either 23- or 25-gauge instruments. Cataract surgery was performed in eyes with a cataract. To begin the PPV procedure, a trocar was inserted at an approximate angle of 30° parallel to the limbus. Once the trocar was past the trocar sleeve, the angle was changed to be perpendicular to the retinal surface. After creating the three ports, we performed PPV using the Constellation® system (Alcon Laboratories, Inc., Fort Worth, TX). Intraoperative scatter photocoagulation was then applied to the retina which results in a complete PRP. We tried to make the same size laser photocoagulation spot by placing the laser probe at the same distance from the retina. The laser power was set to be strong enough to cause a white spot to appear on the retina. The power of the photocoagulation was 150 mW and the duration was 150 ms. Air, 20% sulfur hexafluoride (SF6), or silicone oil was injected into the vitreous at the completion of the vitrectomy to tamponade the retina if needed. We injected 0.3 to 0.5 cc lesser amount of silicone oil than the vitreous volume to avoid postoperative ocular hypertension. After the IOP was adjusted to a normal pressure, the cannulas were withdrawn. The sclera was pressed and massaged with an indenter to close the wound or sutured with 8–0 vicryl to close the wound.

Preservative-free triamcinolone acetonide (MaQaid, 40 mg/vial; Wakamoto Pharmaceutical, Tokyo, Japan) was dissolved in 1 mL of balanced salt solution plus (Alcon Laboratories), and 0.2 mL of the solution was separated and kept in a 1 mL syringe. In the PPV + IVTA group, 0.1 mL (4 mg) was injected into the vitreous cavity through a 30-gauge needle just before the completion of the surgery. In the PPV + STTA group, 0.5 ml (20 mg) TA was injected through the sub-tenon space reaching the posterior pole using a 21-gauge subtenon cannula.

Gentamicin and betamethasone were injected subconjunctivally at the completion of surgery. Anti-inflammatory drops and anti-bacterial drops were administered four times/day for 3 months.

### Statistical analyses

Chi-square tests were used to determine the significance of differences in the categorical data, and independent *t* tests were used to compare normally distributed data. Repeated analysis of variance with post-hoc Bonferroni corrections was used to determine the relationship between the total peripheral thickness and the IOP. Pearson’s correlation coefficient tests and Kruskal-Wallis one-way analysis of variance were used to determine the variables significantly associated with the total peripheral thickness. Multiple linear regression analysis was used to determine the correlation between the total peripheral thickness at 3 days after surgery and the independent variables including the preoperative retinochoroidal thickness, axial length, type of tamponade, operation time, IOP, gauge of surgical instruments, age, surgical type, and sex. A *P* value < 0.05 was considered statistically significant.
